# The Impact of Urbanization on Ecosystem Health in Typical Karst Areas: A Case Study of Liupanshui City, China

**DOI:** 10.3390/ijerph18010093

**Published:** 2020-12-24

**Authors:** Yangling Zhao, Rui Han, Nan Cui, Jingbiao Yang, Luo Guo

**Affiliations:** 1 College of Life and Environmental Sciences, Minzu University of China, Beijing 100081, China; 16054035@muc.edu.cn (Y.Z.); 18301234@muc.edu.cn (R.H.); 2 School of Geography, University of Leeds, Leeds LS2 9JT, UK; gync@leeds.ac.uk

**Keywords:** ecosystem health, land use/land cover, urbanization pressure–state–response model, Liupanshui

## Abstract

The karst region of Southwest China is one of the largest continuous karst areas in the world, and the ecosystem in the karst region is extremely fragile. The city of Liupanshui, a typical karst area in southwestern China, has provided the main energy and raw materials during China’s rapid urbanization in the past few decades. With the continuous deterioration of the environment in Liupanshui and from the viewpoint of sustainable development strategies, research on ecosystem health (ESH) and the assessments of correlations between urbanization and ESH plays an important role in regional sustainable eco-environmental development. Therefore, the impact of urbanization on the ecosystem health of the study area was discussed in this study using a series of remote sensing images and socio-economic data from 1990 to 2015. Studies showed that Liupanshui is undergoing rapid urbanization, and the growth of urbanized land reached a peak between 2010 and 2015. From 1990 to 2015, the level of ESH in Liupanshui trended downward and then increased. During 2000 to 2010, due to the policy of returning farmland to grassland and forestland, the substantial increase in woodland and grassland and the management policy of mining areas have caused a turn in ESH. Although the value of ecosystem health in 2010–2015 increased, the process of urbanization is rapid, so we should pay more attention to the trend in future ecosystem health changes. The findings revealed that urbanization significantly negatively affects the ecosystem health of Liupanshui, and mining has the greatest impact. Therefore, in future urban development, strengthening the management of resource extraction and the supervision of environmental protection, continuing to return farmland to grassland and forestry, and controlling rocky desertification can improve the health of the urban ecosystem in the study area.

## 1. Introduction

Today, according to the World Urbanization Prospects: 2018 Revised Edition of the United Nations Department of Economic and Social Affairs (UNDESA), by 2050, approximately 55% of the world’s population will live in cities, and the global urbanization rate is expected to reach 68% [1]. Urbanization has had many negative effects on ecosystems [2]. For resource-based cities, as the regional economy develops, energy demand increases [3]. However, this not only lead to the reduction of resources, but also affects the health of the ecosystem, through vegetation reduction, biological genetic variation, biodiversity loss, water cycle disturbance, pollution discharge, and land desertification [4]. Ecosystems provide humans with service functions in terms of resources and living environment, and its service function is the cornerstone of human survival and development [5]. Therefore, the impact of urbanization on the ecosystem must be explored.

The karst ecosystem, composed of carbonate rocks, accounts for about 15% of the world’s land area [6,7]. Karst areas have the characteristics of small population capacity, simple community structure, high environmental sensitivity, weak disaster bearing capacity, and difficulty in recovery [8]. Among them, current scholars are focusing on the study of rocky desertification [9]. Rocky desertification marks the collapse of the ecological environment, because it is essentially the top stage of the reverse succession of the karst ecological environment system [10]. In other words, the ecosystem health is fragile in karst areas where there are serious rocky desertification problems, which is a process of land degradation, mainly manifested in soil erosion, bare bedrock, and decreased soil productivity [11,12]. In this fragile ecological environment, population pressure and concentrated land use lead to deeper land degradation, thereby aggravating the vicious circle of ecosystem degradation [13]. Therefore, the research on the ecosystem of the karst area is urgent. From the end of the 1980s to the beginning of the 1990s, Mr. Yuan Dao pointed out the severe ecological problems in the subtropical karst region of southern China, and he initiated the study of karst geomorphology in my country [14]. Many scholars have studied karst areas to explore the causes of rocky desertification from the natural and human factors that induce rocky desertification and provide a theoretical basis for subsequent rocky desertification control [15]. The most prominent conclusion is that increased forest coverage can reduce soil loss. Since 2000, the Chinese government has implemented the eco-engineering policy of returning farmland to grassland and forests, and forcibly combating rocky desertification [16]. Many studies have shown that returning farmland to grassland is effective for ecosystems [17,18]. In recent years, the degree of urbanization in the karst region in Southwest China has continued to increase. Therefore, the impact of urbanization on the sustainable development of regional ecosystems must be examined.

Ecosystem health (ESH) refers to the maintenance of the original state and structure of the ecosystem and the ability to self-regulate and recover after intervention [19,20]. ESH is the most basic prerequisite for maintaining various activities in a region. Its dynamic changes are consistent with human survival. The vigor, organization, and resilience of ecosystems are the core aspects of the ecosystem health concept [21]. At present, the ecosystem health evaluation models include the social–economic–natural composite ecosystem (SENCE), pressure–state–response (PSR) model, ecological footprint, vitality–organization–resilience (VOR), and so on. Among them, the PSR model is logical, easy to operate, and widely used in ecosystem health evaluation [22]. For karst ecosystems, many studies have been conducted on the control of rocky desertification in karst areas [18,23,24]. Current research lacks long-term evaluations and urbanization-related research. Therefore, research on the response of the ecosystem health status in karst areas to urbanization plays an important role in coordinating the sustainable development of geomorphological karst areas. We adopted the PSR model, incorporated rocky desertification sensitivity into the evaluation index system, and uses a 2 × 2 minimum spatial grid to evaluate the impact of urbanization on ecosystem health. In addition, we used different buffer distances to initially explore the impact of mining areas on the ecosystem health of the study area. This was a novel exploration into the ecosystem health and its influencing factors in the study area.

China is the country with the largest karst area in the world, and Liupanshui is the center of the karst geomorphic area in southwest China, with 63.18% of the area belonging to the karst risk area. Moreover, Liupanshui is known as the Southwest Coal Sea, and it is also the second largest economic city in Guizhou Province. Different from other coal areas in China, Liupanshui has a fragile ecosystem with unique karst landforms. The exploitation of resources will inevitably increase the burden on the regional ecosystem, so it is very important to carry out a long-term serial evaluation of the ecosystem health in Liupanshui. Taking Liupanshui as an example, it not only provides a theoretical basis for the assessment of ecosystems in China’s many mineral resource cities, but also covers the assessment objectives of ecosystem health in karst areas. The aims of this study are (1) to ascertain the changes in Land Use/Land Cover and clarify the impact of land use changes on ecosystem health from 1990 to 2015; (2) to evaluate the ecological system health based on PSR model; and (3) to explore the impact of urbanization and mining areas on ecosystem health in order to provide a theoretical basis for regional urbanization development planning.

## 2. Materials and Methods 

### 2.1. Study Area

**Figure 1:**
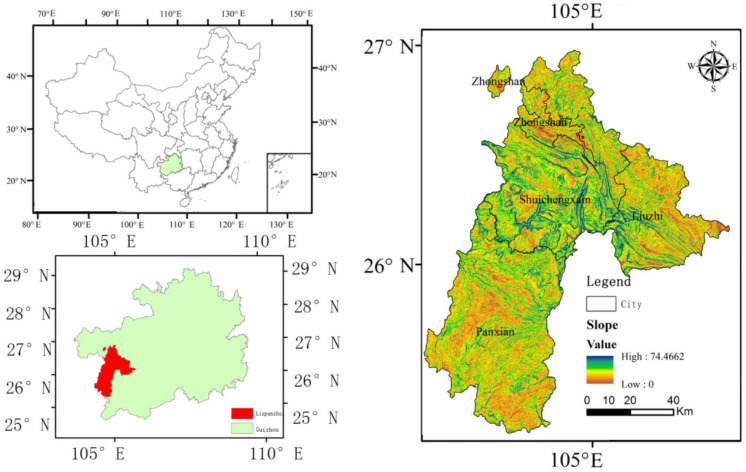
The map of the study area.

The city of Liupanshui, situated between E 104°18’–105°42’ and N 25°19’–26°55’, is located on the slopes of the first and second terraces of western Guizhou and the Yunnan–Guizhou Plateau with a total area of 9965 km^2^ (Figure 1). The Liupanshui belongs to the core area of Southwest Karst and its karst area accounts for 63.18% of the city’s area. The Liupanshui belongs to north subtropical climate type. The elevation ranges from 1400 to 1900 m above sea level. The permanent population of the Liupanshui is 2.859 million. Liupanshui is a representative city in the Southwest Coalfield District, and its raw coal production accounts for 42.07% of Guizhou Province. Coal resources support the economic development of Liupanshui but pose great risks to the regional ecosystem. Therefore, in the future, we should weigh the relationship between economic development and the ecological environment.

### 2.2. Data Collection

The land-use data used for the study area was based on Land Use/Land Cover in the years 1990, 1995, 2000, 2005, 2010, and 2015 which comes from the Chinese Academy of Sciences Resource Environmental Data Center (RESDC). The spatial resolution of land-use dataset is 1000 m × 1000 m. According to National Standard Land-Use Classification of China, land use types in the study area are classified into six primary types and 13 subtypes. The primary classifications are farmland, forest, grassland, water, build and unused land. The comprehensive evaluation accuracy of the first level of land use is more than 93% and that of the second level is more than 90%, which can meet user mapping accuracy demands [22]. The population density and gross domestic product (GDP) per capita data are derived from the Chinese Academy of Sciences with a resolution of 1 km; the digital elevation model (DEM) data are derived from the National Geographic data cloud with a resolution of 1 km. The data of mining area is from coal mine production capacity of Guizhou Province in Guizhou Energy Bureau.

### 2.3. Methods

#### 2.3.1. LULC Change Direction Model

Land use change direction model (LCDM) is a preliminary assessment of the impact of land use change on ecosystem functions [25]. The formula is

(1) $$\mathrm{LCDM}=\frac{\sum_{i=1}^{n}\left[A_{ij}\times \left(D_{j}-D_{i}\right)\right]}{A}\times 100\%$$

where *i* is the *i*-th land-use cover; *j* is *j*-th land-use cover transformed from the *i*-th in a specific period; *A_ij_* is the area of *i*-th land-use cover transforming to *j*-th; A is the total transformed area of all land-use types in the entire study area during this period; and *D_i_* and *D_j_* represent the ecological level of the LULC types before and after transposition (Table 1).

#### 2.3.2. Assessing Ecosystem Health Based on the PSR Model

Based on the grid analysis method, the research area is divided into 2 km × 2 km grid. Based on the PSR model, we select indicators from the three dimensions of pressure, state, and response to estimate the ecosystem health of Liupanshui. Human activities are the greatest disturbance to the ecosystem. We choose population density (POP) and the anthropogenic pressure index (H) to comprehensively assess the pressure from human activities. In addition, in Guizhou where there are many mountains and steep slopes, the downhill farming method is generally adopted, but this method will accelerate the process of soil erosion and rocky desertification on the slope. We can measure the special pressure of karst areas by using the proportion of slope farmland greater than 25 degrees and the land reclamation rate. The status factor refers to the status of the regional ecological environment system, which represents the change trend of the regional ecological environment health caused by natural pressure or man-made pressure. Therefore, this paper selects vegetation coverage, Shannon diversity index (SHDI), Shannon’s evenness index (SHEI) and Contagion (CONTAG) [27], ecological resilience Index (ERI) and ecosystem services (ESV) indicators to comprehensively evaluate the regional ecosystem status. The response layer refers to the countermeasures that human beings make to improve the health of the ecological environment in order to promote the sustainable development of the regional ecological environment. As we all know, the increase in forest coverage can reduce the rate of soil loss. At present, the purpose of returning farmland to grassland is to increase the area of forest, reduce regional soil erosion, and maintain the ecosystem in the region in a healthy state. The rocky desertification means that the karst ecological environment system is in reverse succession, and the ecosystem is becoming more unstable. Using rocky desertification sensitivity indicators can reversely reflect the extent of the effect of ecosystem governance in the region. Therefore, we will choose the proportion of forest land and the sensitivity to rocky desertification as the response layer indicators. Based on the analytic hierarchy process [28], the evaluation system is constructed to determine the weight of each indicator (Table 2).

According to the weight of each individual indicator, the ecosystem health index (ESH) is obtained, which is classified according to the natural segmentation method. The calculation formula of ESH is as follows:

(2) $$\mathrm{ESH}=\overset{n}{\underset{i=1}{\sum }}W_{i}Q_{i}$$

In the formula, ESH is the ecosystem health value, $W_{i}$ is the weight value of the *i*-th single indicator, and $Q_{i}$ is the normalized value of the *i*-th single indicator. Among them, each index must be normalized, and the formula is

(3) $$Q_{i}\;=\;\left(X_{i}-X_{\min}\right)/\left(X_{\max}-X_{\min}\right)$$

(4) $$Q_{i}=1-\left(X_{i}-X_{\min}\right)/\left(X_{\max}-X_{\min}\right)$$

In the formula, (3) is the positive index processing formula, (4) is the negative index processing formula, $Q_{i}$ is the normalized value, $X_{i}$ is the actual value of the index, $X_{min}$ is the minimum actual value of the index, and $X_{max}$ is the actual value of the index Maximum value.

#### 2.3.3. Mapping Urbanization

The level of urbanization consists of four stages. Population growth is the most important feature of modern urbanization; economic development is the significance of urbanization; the increase in construction land is the expansion of the above factors; and the standard of living is the final result of urbanization [29]. Therefore, this study will select population density (POP), per capita income (GDP) and construction land ratio (CLP) to measure the level of regional urbanization [30,31]. Indicators of POP, GDP, and CLP are integrated into the comprehensive level of urbanization [32]. Standardize the indicators and take the average of the normalized data sum.

#### 2.3.4. Correlation Analysis between Ecosystem Health and Mining Area

**Figure 2:**
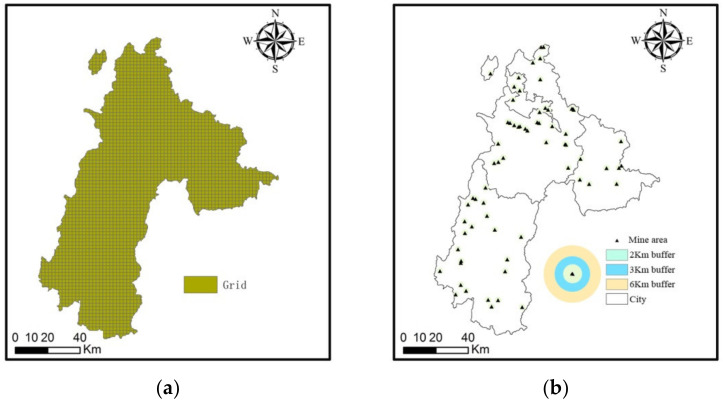
(**a**) The range of Liupanshui (**b**) Mining area and buffer zone in Liupanshui.

To further analyze the spatial characteristics of ecosystem health, the ecosystem health of 66 typical mining areas in Liupanshui in 1990, 1995, 2000, 2005, 2010, and 2015 was explored. In ArcGIS, 2 km, 3 km, and 6 km buffer zones are established for all representative mines to merge [33] (Figure 2). Among them, the 0–2 km mine point buffer has a relatively low overlap, and its data has a precise meaning. Then use the hydrological analysis module of ArcGIS 10.4 to extract information about the mine. In addition, in ArcGIS, the ecosystem health level of each mining area is counted.

## 3. Results

### 3.1. Analysis of Spatial and Temporal Change of the Land Use Pattern in Liupanshui

From 1990 to 2015, the dynamics of land use in Liupanshui changed considerably. The single land use types of construction land and forest land increased, while grassland and cultivated land showed a downward trend. From 1990 to 2015, the forest floor area increased by 53 km^2^, which is distributed in the southwest of Shuicheng County and the southwest of Pan County. Faer Ecological Park has fruit trees and the ancient ginkgo tree scenic park. The construction land area increased by 66 km^2^ and its dynamic value is the highest. It is distributed in the north and southwest of Panxian County, Zhongshan District, and the border with Shuicheng County. Among them, Zhongshan District is the most constructed area, indicating that the degree of urbanization in Zhongshan District is higher than in other regions. The area of arable land and grassland decreased by 43 and 76 km ^2^, respectively. There was no change in the water bodies.

To clarify the impact of changes in land use types on ecosystems, we conducted a preliminary assessment of the impact of changes in land use types on ecosystem health based on the land use/land cover (LULC) model changes. According to the formula, we obtained LCDM_1990–2000_ = 0.13% and LCDM_2000–2015_ = 4.53%. The results showed that the LCDM value increased from 1990 to 2015, indicating that the changes in land use types from 1990 to 2015 had a beneficial effect on the ecosystem function of the study area.

### 3.2. Spatio-Temporal Change Analysis of Ecosystem Health Elements in Liupanshui 

**Figure 3:**
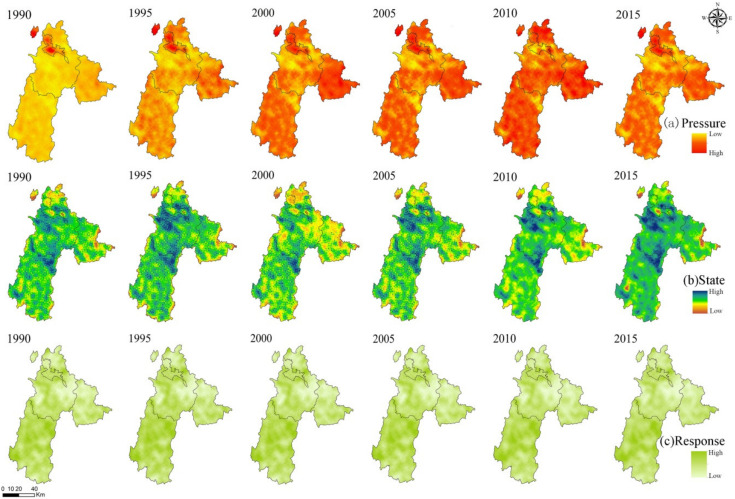
Temporal and spatial distribution of each index layer from 1990 to 2015: (**a**) Pressure; (**b**) State; (**c**) Response. (According to each color band, we can know the changing trend of the value of different index layers).

The index layer of regional ecosystem pressure is shown in Figure 3. The pressure value is composed of indicators such as population density, reclamation rate, and the proportion of farmland sloping more than 25°. The smaller the pressure value, the greater the pressure on the ecosystem. From 1990 to 1995, the pressure value in Zhongshan District was the highest, and other areas were under low pressure. From 1995 to 2005, Liuzhi Special Zone and the northern part of Shuicheng County changed from moderate to high-intensity pressure, while pressure in other areas of Shuicheng County and Pan County was at a reasonable level. From 2010 to 2015, the pressure value in Liupanshui increased, mainly because the pressure state in Pan County increased to high and the areas with low pressure gradually decreased.

The status layer is the criterion layer that characterizes the status of the regional ecological environment. When the status value is higher, the regional ecological environment is in good condition. From 1990 to 2015, the overall state value of Liupanshui rose from 0.247 to 0.256. The status value of the middle of the city were higher. The state values of the northern, eastern, southern, and enclave areas of the city were low, indicating that the state of the above-mentioned areas was poor and needs to be strengthened.

According to the environmental problems and corresponding environmental protection measures in Liupanshui, the response layer of the study area was characterized by forest land and rock desertification sensitivity. The results are shown in Figure 3. From 1990 to 2015, the response index value of Liupanshui tended to be stable, and the regions with lower response values were mainly in the east and west of Liuzhi and Shuicheng County. In the whole study area, the descending order of response values are Panxian, Zhongshan District, Liuzhi, and Shuicheng County.

### 3.3. Temporal and Spatial Changes in Ecosystem Health in Liupanshui

**Figure 4:**
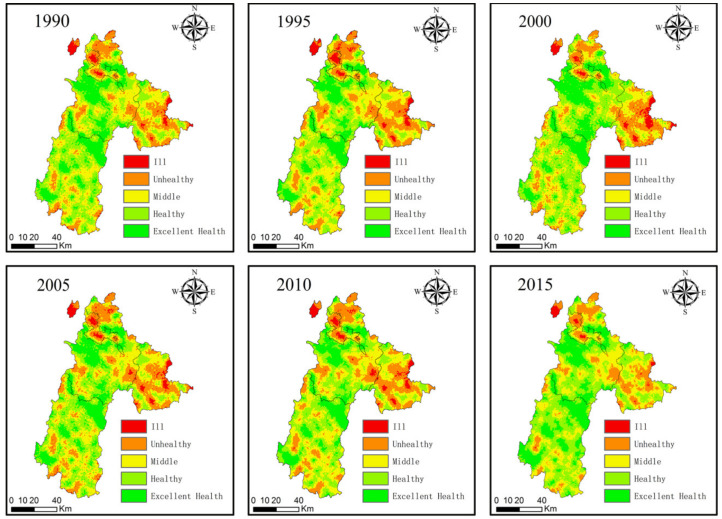
Spatio-temporal distribution of ecosystem health in Liupanshui.

According to the natural segmentation method, the ecosystem health value of the study area was divided into five categories: excellent healthy, healthy, middle, unhealthy, and ill. The results of the spatial and temporal distribution of the regional ecosystem health are shown in Figure 4. The ESH level in the Northwest Liupanshui was higher than in the south. The ESH in the eastern region has changed significantly, while the health of the ecosystem in the central region was relatively stable.

From 1990 to 2015, the worst areas of Liupanshui’s ecological environment health were mainly distributed in Zhongshan District and the eastern part of Liuzhi, and their proportion decreased from 3.29% to 1.11%. The ecosystems in Northern Shuicheng County, Liuzhi, and Southern Pan County were in an unhealthy state, and their proportions showed downward trends, decreasing from 19.93% to 12.94%. The above-mentioned regions have poor ecosystem health and are key areas for future ecological health management. The eastern part of Shuicheng County and Pan County were unhealthy, which are located around unhealthy areas and the risk of deterioration is higher. The ecological system in the central part of the city continued to be healthy or above, with various nature reserves in the city, such as Yushe National Forest Park, Wumeng Prairie, and Wild Black Monkey Nature Reserve. In short, Pan County has the healthiest ecosystem, followed by Shuicheng County and Liuzhi. However, the ecosystem health in Zhongshan District is the worst.

### 3.4. Ecosystem Health and Urbanization Response in Liupanshui 

According to the results of the six-period global bivariate Moran index (Table 3), we found an obvious negative correlation between ecological environment health and urbanization in Liupanshui. To test whether Moran’s I was valid, Monte Carlo was used to simulate 999 tests in Geoda. All results passed the significance test at 0.001, indicating that the spatial autocorrelation was significant with a confidence of 99.9%. From 1990 to 2015, Moran’s I index declined, indicating that urbanization was having an increasingly negative effect on the health of the ecosystem. From 2000 to 2015, Moran’s I steadily increased, indicating the negative effects of urbanization on the ESH have weakened.

**Figure 5:**
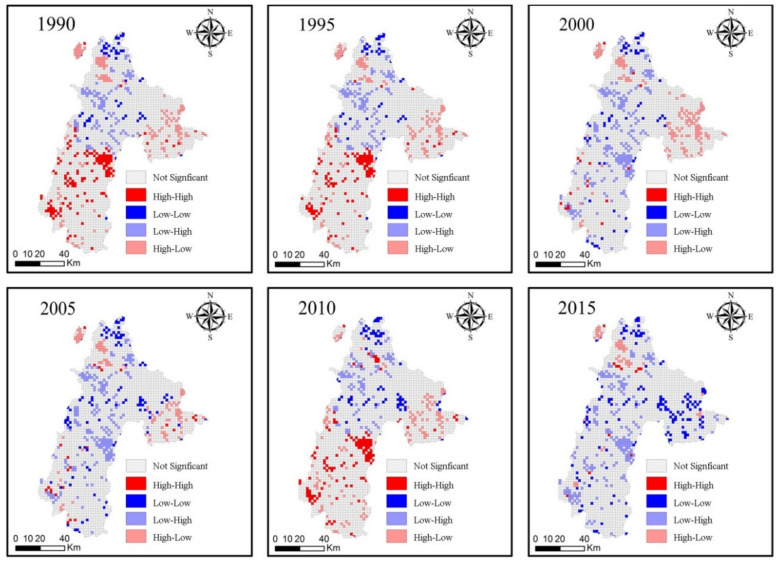
LISA cluster maps between ecosystem health (ESH) and urbanization level; High–High: High Urbanization and High ESH; Low–Low: Low Urbanization and Low ESH; Low–High: Low Urbanization and High ESH; High–Low: High Urbanization and Low ESH.

According to Figure 5, we identified four different spatial correlations between urbanization level and ecosystem health in the study area: high and high aggregation, high and low aggregation, low and high aggregation, and low and low aggregation. From 1990 to 2000, the number of low- and high-aggregation grids of Urbanization and ESH increased, and they were distributed in the west and northeast of Shuicheng County and the northeast of Panxian County. The number of high- and low-aggregation grids of Urbanization and ESH tended to be stable, distributed in Zhongshan District and Liuzhi. From 2000 to 2015, the number of low- and high-aggregation grids of Urbanization and ESH decreased slightly. The number of high- and low-aggregation grids of Urbanization and ESH showed a downward trend, only distributed in Zhongshan District and Liuzhi. Overall, Zhongshan District has a higher degree of urbanization and the greatest impact on the ecosystem, while Liuzhi has a lower degree of urbanization.

### 3.5. Ecosystem Health and Mining Area Response in Liupanshui 

From 1990 to 2015, the health of the Liupanshui mining site ecosystem was considerably reduced from 8 to 2, and the unhealthy mining sites fluctuated from 27 to 24. From 1990 to 2010, unhealthy mining sites increased from 14 to 23. In 2015, it was reduced to 17. This region is at the zero boundary of ecosystem health, where environmental management should be strengthened to prevent further deterioration. In addition, the slow growth of healthy mining areas increased from 18 to 20, indicating that the ecosystem health of mining areas in the city is gradually improving.

Through the spatial buffer analysis within 1–6 km around the mining sites, and comparing the ecosystem health values of each buffer, a 2 km buffer zone in Liupanshui mining area was selected for analysis. The results are shown in Table 4. From 1990 to 2015, the pressure value and status of the mining area showed a downward trend and then increased, indicating that the pressure in the mining area first increased and then decreased and recovered after the state worsened. The response value showed a downward trend from 1990 to 2015, indicating that the response to environmental protection in the mining area was relatively weak. The ecosystem health in 1990–2015 showed a downward trend and then increased. The health of the ecosystem in the mining area gradually recovered after 2000, indicating that the environmental protection measures implemented in the mining area in 2003 and the mergers in the mining area in 2011 had a significant effect on the health of the regional ecosystem. Compared with the whole city, the pressure, status, response, and ecosystem health values of the mining area from 1990 to 2015 were lower than the overall level of the city, indicating that the mining areas have impacted the surrounding ecosystem.

## 4. Discussions

### 4.1. Spatial-Temporal Characteristics of ESH

Based on the PSR model and the actual situation in Liupanshui, we selected appropriate evaluation indicators to build an ecosystem health model to obtain the ecosystem health value of Liupanshui from 1990 to 2015. The results showed that the health of the Liupanshui ecosystem showed a downward trend after 1990–2015, and that the regional ecological environment gradually recovered after 2000. The degree of urbanization in the study area is increasing each year, and the population and the area of arable land have increased, thereby increasing the pressure on the study area, resulting in a decline in the health of the regional ecosystem. However, although urbanization is ongoing, the ESH in the study area gradually recovered after 2000, mainly because of returning farmland to grassland and forestry and environmental protection in mining areas. Due to various water and soil erosion control projects, the area of forest land increased, and the area of cultivated land decreased, which increased the value of the status and response layer. Because these indicators are strongly influenced by forest, the health of the ecosystem in the region eventually changed, which is consistent with the results of the ecological restoration studies in Guangxi [34] and Bijie [25]. In addition, from 2010 to 2015, construction land increased in a large area and the increase rate of regional ecosystem health slowed. According to the spatial changes in ecosystem health, the spatial distributions of population density and land use are closely related to regional ecosystem health. Due to the topography and natural resources of different regions, the degree of urbanization in different regions varies widely. The northwest and the easternmost part of the study area belong to urban- and county-level administrative centers with high population density and numerous construction sites, resulting in a low ESH in this area. The north, east, and south of the city are in an unhealthy state, accounting for an area of about 12.9%. This area belongs to the mining economic development zone of Liupanshui. However, in the early days of the founding of the People’s Republic of China, the lagging technology and lack of awareness of environmental protection caused damage to the ecosystem’s functioning. Therefore, the ecosystem in this area is fragile and has easily deteriorated into an unhealthy state. It is a key that needs management. The ecological system in the central and northeastern parts of Liupanshui are in good condition because of the relatively good ecological environment in mountainous areas and the natural reserves in areas with low levels of human activity [30]. In addition, the central and southern regions are key areas for returning cultivated land to grassland and forest, gradually changing the ecosystem from unhealthy to healthy. Therefore, in future urban development, it is necessary to strengthen the supervision of environmental protection in the study area.

### 4.2. Factors Influencing ESH

As a typical mining resource city in a karst area, Liupanshui is experiencing both the serious natural problem of rocky desertification and rapid urbanization. The ecosystem in the study area is influenced by many factors. We analyzed the impact of ecosystem health from the perspective of urbanization and returning cultivated land to grassland and forest and selected the representative mining industry for further discussion to provide suggestions for the future urban development of the region.

We selected three indicators, POP, CLP, and GDP, of urbanization level and ecological environment health as dependent variables and used Geoda software to determine the spatial relationship between ecological environment health and urbanization. The results showed that a significant negative correlation existed between urbanization and ecosystem health, and the effect of urbanization on the ecosystem was gradually increasing. However, in 2010, the impact of urbanization on the health of the regional ecosystem was extremely low. The reason was that the snow disaster and drought in Guizhou province negatively impacted the regional economy, which led to a low degree of urbanization. The northwest, east, and few southern regions of the city belong to administrative function areas. The urbanization indicators, such as population density and GDP, in the region were higher than those in the surrounding areas. Therefore, urbanization in this area had a large impact on the health of the ecosystem. Conversely, the central area of the city had a lower degree of urbanization, and it had better ecosystem health. The low degree of urbanization in the northern part of the city and the low ecosystem health value indicated that the factors affecting this area were not urbanization, but other factors, such as topographical factors. Therefore, appropriate environmental protection policies and planning schemes should be adopted in different regions.

The development of coal resources will inevitably have a destructive effect on the ecosystem. We explored the ecosystem health within 2 km of a typical mining area, and the results showed that the ecosystem health in the buffer zone showed a downward trend during 1990 to 2010, but it gradually recovered in 2015. In addition, the ecosystem health value in the buffer zone was lower than the surrounding value. The above phenomenon mainly occurred due to the mining area merger project in 2003, which reduced the pollution of small and technologically lagging mining areas and promoted the gradual restoration of the ecological system of the mining area.

### 4.3. Suggestions

City planners and environmental managers are facing the problem of balancing the relationships between urban construction, economic development, and ecological protection. At present, some solutions have been proposed, but some problems remain, such as the lack of urbanization, in managing the ecological environment in karst areas. We provide some suggestions for the construction of resource-based cities in the Karst region.

From the changes in the ecosystem health value of Liupanshui in the past 25 years, the regional ecosystem health is currently slowly recovering, but it has not recovered to its 1990 state. Therefore, as the degree of urbanization gradually increases, it is necessary to continue to strengthen the management of mining areas and the supervision of ecological projects that are returning farmland to grassland and forests to ensure the continued healthy restoration of the ecosystem in the region.

For the administrative regions of Liupanshui, it is necessary to control the increase in population and construction land to reduce the ecosystem pressure in the region. In addition, the proportion of green coverage in the region should be increased and a green-friendly city should be built. In the mining economic parks in the north and southeast of the study area, managers should reduce the number of mines with lagging technology and low output. The managers should strengthen the inspection of the mine and the surrounding environment to avoid the impact of waste residues on the environment after production.

In the study area, farmland should be returned to grassland and forests, the supervision of the implementation and subsequent maintenance of returning farmland to grassland and forests should be further increased to ensure the significance of the ecological projects in the region.

For natural mountain areas in the study area, such as the central part of the city, when using natural resources to develop tourism, the ecological environment of the protected area should be placed first to ensure the natural state of the study area.

## 5. Conclusions

In this study, we selected multiple indicators to measure the change of ecosystem health in Liupanshui City from 1990 to 2015, and we explored the spatial correlations between urbanization and ecosystem health by heterogeneity and hotspot analysis. The transfer matrix was used to explore the changes of Land use/Land cover (LULC). LULC change direction model (LCDM) was used to preliminarily assess the impact of LULC changes on the ecosystem. The results showed that the main changes in LULC are the increase in forest and construction land and the decrease in farmland and grassland. The changes in LULC have positively developed the ecosystem function, indicating that returning farmland to forests has had a certain effect on restoring the health of the regional ecosystem. However, after 2010, the area of construction land increased significantly. Second, we explored the spatial heterogeneity of ESH by hotspot analysis. The ESH in the middle of the study area is good because it contains a nature reserve with low human activity. The northern, eastern, and northwestern parts of the study area have low ESH and are key areas for future improvement. Due to human pressure and protection engineering, the ecosystem health value in the study area declined and then increased. In the earlier stage (1990–2000), the decline in ESH was related to the rapid urbanization in the region, especially the development of mining areas. In the final stage (2000–2015), the increase in regional ESH is related to the increase in the area of forest. In the middle of the study area, the growth rate of ESH in 2015 was relatively slow, mainly related to the urbanization of the region. Finally, according to the results of the local binary analysis, we found that the area has been affected by urbanization, which can be used to formulate corresponding urban management policies. In recent years, however, it can be seen that the policy of developing new tourism, has led to more human intervention in the mountains. More attention should therefore be paid to achieving a high degree of integration of ecological, economic, and social benefits while at the same time maintaining and consolidating the existing ecological benefits, to achieve sustainable development of the regional economy.

## Figures and Tables

**Table 1:** Ecological levels of the different land use [26].

LULC	Farmland	Forestland	Shrubland	High-Density Grassland	Mid-Density Grassland	Low-Density Grassland	Water	Unused Land
Ecological levels	0.11	0.245	0.147	0.125	0.063	0.018	0.282	0.01

**Table 2:** Index system of the ecosystem health evaluation and their weights.

Target Level	Standard Level	Feature Level	Indicator Level	Weight
Ecosystem Health (ESH)	Pressure	Population	Population Density	0.11
			Man-made interference	0.14
		Resources	Land Reclamation Rate	0.12
			25 proportion of sloping cultivated land	0.05
	State	Vigor	Vegetation Coverage Index	0.11
		Organization	Shannon’s Evenness Index	0.03
			Shannon Diversity Index	0.03
			Contagion	0.03
		Resilience	Resilience	0.10
		Contribution	Ecosystem Service Value	0.13
	Response		Proportion of forest land	0.10
			Sensitivity of rocky desertification	0.05

**Table 3:** The result of Moran’s I.

	Year	1990	1995	2000	2005	2010	2015
Urbanization	Moran’s I	−0.127	−0.138	−0.256	−0.161	−0.031	−0.149
	z-Value	−11.64	−12.56	−22.70	−15.07	−3.012	−13.48
	*p*-Value	0.001	0.001	0.001	0.001	0.001	0.001

**Table 4:** Ecosystem health of the buffer.

Time	0–2 km Buffer				0–3 km Buffer			
	P	S	R	ESH	P	S	R	ESH
1990	0.323	0.247	0.072	0.643	0.329	0.246	0.072	0.643
1995	0.321	0.240	0.071	0.632	0.321	0.240	0.071	0.632
2000	0.314	0.229	0.071	0.616	0.314	0.229	0.071	0.616
2005	0.325	0.241	0.071	0.638	0.325	0.241	0.071	0.638
2010	0.315	0.242	0.067	0.623	0.315	0.242	0.067	0.623
2015	0.336	0.251	0.067	0.654	0.336	0.251	0.067	0.654
